# DengueME: A Tool for the Modeling and Simulation of Dengue Spatiotemporal Dynamics †

**DOI:** 10.3390/ijerph13090920

**Published:** 2016-09-15

**Authors:** Tiago França Melo de Lima, Raquel Martins Lana, Tiago Garcia de Senna Carneiro, Cláudia Torres Codeço, Gabriel Souza Machado, Lucas Saraiva Ferreira, Líliam César de Castro Medeiros, Clodoveu Augusto Davis Junior

**Affiliations:** 1 Departamento de Computação e Sistemas (DECSI), Instituto de Ciências Exatas e Aplicadas (ICEA), Universidade Federal de Ouro Preto (UFOP) - Campus João Monlevade, João Monlevade, MG 35931-008, Brasil; gabrielmchdo@gmail.com (G.S.M.); saraivalucas12@gmail.com (L.S.F.); 2 Programa Pós-Graduação em Epidemiologia em Saúde Pública, Escola Nacional de Saúde Pública Sérgio Arouca (ENSP), Fundação Oswaldo Cruz (Fiocruz), Rio de Janeiro, RJ 21045-900, Brasil; raquelmlana@gmail.com; 3 Departamento de Computação (DECOM), Instituto de Ciências Exatas e Biológicas (ICEB), Universidade Federal de Ouro Preto (UFOP) - Campus Morro do Cruzeiro, Ouro Preto, MG 35400-000, Brasil; tiago@iceb.ufop.br; 4 Programa de Computação Científica (PROCC), Fundação Oswaldo Cruz (Fiocruz), Rio de Janeiro, RJ 21045-900, Brasil; codeco@fiocruz.br; 5 Instituto de Ciência e Tecnologia, Universidade Estadual Paulista Júlio de Mesquita Filho (UNESP), São José dos Campos, SP 12247-004, Brasil; liliam.medeiros@ict.unesp.br; 6 Departamento de Ciência da Computação (DCC), Instituto de Ciências Exatas (ICEx), Universidade Federal de Minas Gerais (UFMG), Belo Horizonte, MG 31270-010, Brasil; clodoveu@dcc.ufmg.br

**Keywords:** modeling, simulation, dengue, *Aedes aegypti*, framework, spatiotemporal, model, DengueME

## Abstract

The prevention and control of dengue are great public health challenges for many countries, particularly since 2015, as other arboviruses have been observed to interact significantly with dengue virus. Different approaches and methodologies have been proposed and discussed by the research community. An important tool widely used is modeling and simulation, which help us to understand epidemic dynamics and create scenarios to support planning and decision making processes. With this aim, we proposed and developed DengueME, a collaborative open source platform to simulate dengue disease and its vector’s dynamics. It supports compartmental and individual-based models, implemented over a GIS database, that represent *Aedes aegypti* population dynamics, human demography, human mobility, urban landscape and dengue transmission mediated by human and mosquito encounters. A user-friendly graphical interface was developed to facilitate model configuration and data input, and a library of models was developed to support teaching-learning activities. DengueME was applied in study cases and evaluated by specialists. Other improvements will be made in future work, to enhance its extensibility and usability.

## 1. Introduction

Dengue incidence increased rapidly in the last two decades [1,2,3]. Some estimates suggest that almost 390 million new infections occur each year [3], and about half of the world’s population is at risk [2]. However, problems with under-reporting and case misclassification suggest that the full impact of the disease is unknown and that new approaches for surveillance are required [4]. More recently, the emergence of other arboviruses, such as chikungunya [5] and zika [6], particularly in South America, have posed new challenges for surveillance and control.

Dengue is a viral infection transmitted between humans and mosquitoes, with four serotypes that are rapidly spreading worldwide [4]. Until recently, its primary vector, the *Aedes aegypti* mosquito, which is well adapted to urban areas, was distributed mainly across tropical and subtropical regions [4,7]. However, now, it has spread to North America and Europe [8]. A secondary vector, *Aedes albopictus*, also expanded its geographic range in recent years [8,9]. The risk of dengue outbreaks and endemicity occurs mainly in tropical and subtropical regions [3]. However, the disease is spreading to North America and Europe [10,11,12], due to the presence of *Aedes* and the introduction of the virus.

Dengue transmission is a complex process that involves the interaction of multiple agents (humans, mosquitoes and virus) in a heterogeneous space. The space itself is complex enough to provide many challenges for dengue transmission studies. The current pandemic was favored by a combination of several factors [4]. These included the global movement of hosts and vectors (that speed up virus circulation [13,14,15]), urban crowding (which favored multiple transmissions by a single infectious mosquito) and loss of effect of previously efficient vector control strategies [16]. To map and describe global patterns of vulnerability to dengue transmission, some researchers have created risk maps and estimated the global distribution of dengue from notification and environmental data [3,17,18,19,20]. Although knowledge about the global situation of the disease is essential, it is also important to understand its dynamics at a local scale, where the encounters between vectors and hosts happen.

Microclimate conditions, such as temperature, rainfall and humidity, interfere with all vector development stages, from egg viability to adult longevity and dispersal, among other aspects of dengue transmission [21,22,23,24,25].

Sociodemographic and environmental factors that contribute to increased dengue incidence [26,27] include: unplanned urbanization, high human population density [26,28,29,30,31], as well as the precariousness of garbage collection and water supply services, which favor the proliferation of mosquito breeding sites [32,33]. Although *Ae. aegypti* generally flies short distances during its life [34,35], air and land transportation systems promote the spreading of viruses and vectors and allow fast displacement of infected people and vectors between different regions [29,36].

Despite efforts to develop a vaccine [37,38,39], as of mid-2016, there is only one licensed product (Dengvaxia^®^, CYD-TDV, Sanofi Pasteur, Lyon, France), which has shown only moderate levels of protection and is recommended only to individuals above nine years of age. Other candidates are in different stages of development and evaluation [40,41]. However, none of them can be an immediate global panacea [4]. Besides, a dengue vaccine does not prevent the spread of zika and chikungunya viruses. Therefore, improvements in treatment and innovative approaches to understand and prevent transmission should continue. Several other control strategies have been proposed, studied and tested. They include reducing the abundance of the vector [42], introducing genetically-modified mosquitoes [43], infecting vectors with other pathogens [44,45,46,47] and preventing mosquito-human contact [48]. An improved understanding of the current epidemiology of the disease and its potential for future spread can assist policy makers in allocating resources to face this global public health challenge [4].

Several dynamic models have been developed to describe dengue transmission dynamics in space and time [49,50,51,52] and the behavior of its primary vector, *Ae. aegypti* [8,53,54,55,56,57,58,59,60]. They are fundamental tools for comparing different control methods and can evaluate what-if scenarios, but creating computational models accounting for several populations (metapopulation models), heterogeneity (e.g., variations in susceptibility and in responses to infection) and spatial heterogeneity requires great expertise and advanced programming skills.

This work presents the design and current development stage of the Dengue Modeling Environment (DengueME) framework. DengueME is an open source tool that supports the development of spatiotemporal models for simulating dengue and its vector, integrated with a Geographic Information System (GIS). The software and its source code are available at the website <http://github.com/ufopleds/DengueME>, under a BSD 2-clause license. The pre-compiled software is available for Windows, and source code is available for users to compile in Linux and OS X.

DengueME was developed as a tool to assist the design of site-specific and vector population-specific control strategies for dengue. To reach this goal, DengueME provides: (i) a collection of built-in models that can be combined to represent various scenarios; (ii) a modeling language that accommodates both compartmental models and individual-based models; (iii) a user-friendly graphical interface for easy model selection and configuration; (iv) a flexible and extensible architecture to allow contribution from users; and (v) a standard format for data exchange to allow communication between DengueME models.

## 2. Related Work

Since the emergence of Ross–Macdonald’s quantitative theory for understanding and measuring mosquito-borne pathogen transmission and control [61], many mathematical models have been developed and applied to study the dynamics of mosquito-borne diseases, mainly malaria, dengue, West Nile virus and filariasis [62].

**Figure 1:**
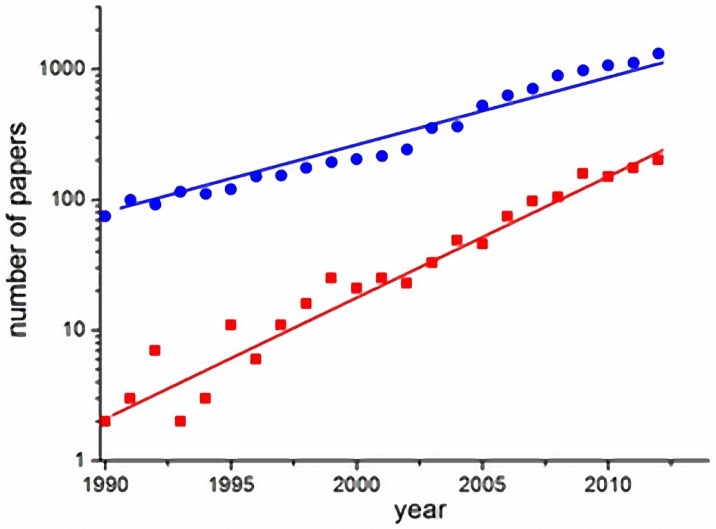
Exponential growth of publications on dengue (blue circles) and specifically on dengue models (red squares) in the last two decades (search done in the ISI-Web of knowledge database) [63].

Computational models are useful for understanding the determinants of disease transmission processes and to analyze the impact of control strategies. Figure 1 shows the exponential growth of the number of scientific articles on dengue in general (blue circles) and articles on dengue models (red squares), in the last two decades (source: ISI-Web of Knowledge). The number of articles on dengue models has grown faster than the number of articles on dengue in general. The increased interest in modeling shows the potential demand for frameworks that feature fast implementation, selection and testing of alternative dengue models in a single environment.

A systematic review of mathematical models of mosquito-borne pathogen transmission, conducted by Reiner et al. (2013) [62], shows an increasing recognition of geographic, ecological and epidemiological complexities in modeling transmission. Models increasingly include more aspects of the pathogen, vector, host populations and their encounters, as well as external drivers of transmission and control of the disease. Among the several external drivers that affect transmission, temperature is one of the most important. Although models have incorporated temperature dependence into specific components of the vector’s life cycle, the most common approach considers the emergence of adults from aquatic habitats as a parameter, with seasonal forcing, but ignoring the aquatic-phase dynamics. Researchers argue there is a need to develop better models of mosquito population dynamics, including the ecology in their aquatic habitats [62]. More recent work has begun to address these aspects [57,59,64].

Often, however, models lack a good description of variability within populations (host and vector), particularly their spatial and temporal heterogeneity [62]. These deficiencies arise in part from the difficulty in obtaining and producing adequate data to parameterize the models. Sometimes, the data are available on a different scale from that required for the study, and extrapolations could introduce errors and bias [27].

The theoretical framework developed for malaria by Ross and Macdonald [61] is evolving with the emergence of new approaches that incorporate heterogeneity, the ecological and social context in which mosquito blood feeding occurs, the mobility of hosts and mosquitoes and the use of proper scales to measure the transmission and to model the dynamics and control of diseases [65]. We argue for a tool that allows building pedagogical, theoretical and empirical models of dengue transmission and vector ecology, using different scales and different paradigms, with support for the use of real data, ranging from simple time series, such as daily temperatures, to more complex urban geographic data. As indicated by Messina et al. (2015) [27], there is a consensus about the influence of climate and environmental variations in the global distribution of dengue. Therefore, models should consider these aspects.

Models have other uses beyond prediction [66]. Modeling and simulation are used as a tool to support planning, decision making and intervention assessments in public health. Models based on system dynamics have been applied in public health since the 1970s, in areas such as disease epidemiology and healthcare capacity planning [67]. Agent-based modeling (ABM) allows describing structures and behaviors at smaller scales and observing the emergent dynamics at larger scales. ABM is also used to study transmissible and non-transmissible diseases [68]. Other methods such as network analysis are also applied [69,70,71,72]. Simulations allow a more extensive investigation of alternative scenarios of spatial spread and its relationship to multiple influencing factors. They can also explore the feasibility of control strategies and support the implementation and evaluation of alternative intervention and control methods. According to Knight et al. (2016) [73], results from models can be linked to public health policy in at least three ways: (i) “improve our understanding of infectious disease epidemic systems so that public health practitioners can better target key drivers of epidemic spread”; (ii) “evaluate and compare the potential epidemiological and economic impact of alternative public health interventions”; (iii) “reveal data gaps that, if filled, would enable public health officials to make more evidence-based decisions in the future”. Further discussions and applications can be found in [73,74,75,76,77].

Some initiatives support users on studying environmental phenomena through computer modeling and simulation. Examples include platforms that allow model building through interaction with graphical interfaces and the usage of visual metaphors (e.g., diagrams) [78,79,80,81]. There are generic frameworks for modeling globally the spread of diseases, such as Spatiotemporal Epidemiological Modeler (STEM) [82] and Global Epidemic and Mobility Model (GLEaM) [83], or using connected networks, such as EpiFire [84]. There is also a graphical interface software developed to support the use of an intervention model for *Plasmodium falciparum* malaria [85]. However, we did not find freely-available solutions dedicated to dengue modeling and targeting local dynamics at intra-urban scales.

## 3. DengueME Framework Overview

DengueME is a software framework for modeling and simulating the spatiotemporal dynamics of dengue and its vector. It provides services to support the development of integrated models and the evaluation of intervention scenarios. The following section presents DengueME’s requirements, design and current development status.

### 3.1. Framework Requirements and Design

As discussed earlier, a framework for dengue modeling should offer a basic library of epidemiological (disease transmission) and entomological (vector population dynamics) spatiotemporal models. Users must be able to configure and parameterize these models according to their needs, driven by the intended application (from pedagogical to real case studies). The current version of DengueME includes models developed by Medeiros et al. (2011) [51] and Lana et al. (2011 and 2014) [56,86], adapted to allow easy integration with a geographic database. In DengueME, models can be developed using multiple scales and modeling paradigms, including differential equations, agents, cellular automata and hybrids. Models are implemented using the high level programming language TerraML (Terra Modeling Language) from TerraME [87]. Currently, DengueME is an extension of TerraME, a platform for modeling and simulating environmental systems.

DengueME models can be directly parameterized and configured using the TerraML programming language. For users with little or no programming experience, DengueME provides a friendly Graphical User Interface (GUI) for customization of models and the design of scenarios. A Visual Development Environment (VDE) allows users to select and integrate sub-models (modules) from the framework’s library, parameterizing them using data from geographic databases or files containing time series and tabular data.

A dengue modeling framework should be continuously updated, in a collaborative development. DengueME accepts new models to its library as a module (or a black-box). Modules contain TerraML code that implements the model and an XML file that describes its parameters (e.g., type and format), inputs and outputs.

Model building is the act of simplifying reality by isolating elements and associations considered relevant to the problem at hand. Modeling is an iterative process involving a sequence of steps, divided into activities and performed with the help of specialized tools. Some general steps are necessary to make the models methodologically acceptable. These include defining their purpose and objectives, conceptual design, implementation and tests, sensitivity analysis, calibration, validation and scenario analysis. The DengueME framework helps users in some of these modeling activities. For instance, its graphical interface assists users in specifying models and analyzing scenarios, helping with activities such as model parameterization, execution and the presentation of simulation results. Some resources to support users through the GUI in other steps, like calibration and validation, are still under development. A detailed presentation on how to build a model is beyond the scope of this article, but additional information about this topic can be found in [88,89,90,91,92,93,94,95].

**Figure 2:**
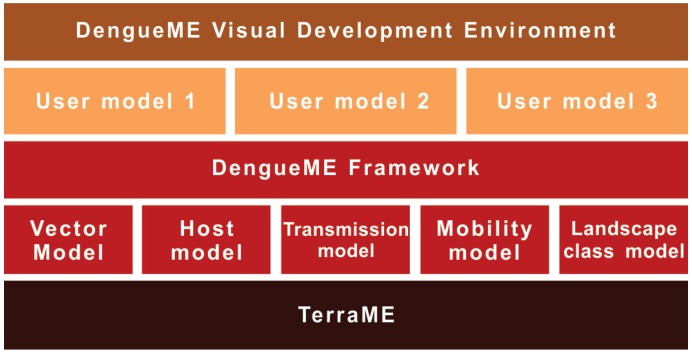
DengueME architecture [63].

Figure 2 presents an overview of the DengueME framework architecture. In the lower layer is the TerraME platform for environmental modeling, used to build the models. The second layer is the DengueME framework, composed of a set of models and middleware to integrate them. Next, there is a layer formed by the models and scenarios customized by the user. They can be developed using the DengueME VDE, available at the top layer of the architecture.

The models and framework layer are developed using TerraML, a modeling language provided by the TerraME platform. TerraML is a script language that extends the Lua programming language, offering an Application Programming Interface (API) designed to support the modeling and simulation of environmental phenomena. DengueME VDE was developed using C++, Qt (an open source user interface library) and XML. Modelers only have to know TerraML, currently the only modeling language supported by the framework, to create new models. The user interface of new models can be directly created using DengueME VDE, with no additional programming. Model users need to interact only with the graphical interface to parameterize models, create scenarios and execute simulations.

### 3.2. Models

The dynamics of dengue transmission is governed by a complex interaction between humans, mosquitoes and viruses, on a heterogeneous landscape. The modularization of this dynamics into independent components allows the user to work on different levels of detail and complexity, according to his or her goals. The following sections present the model components of DengueME (Figure 2).

#### 3.2.1. Vector Models

The vector (entomological) model can be represented as a system of differential equations that describe *Ae. aegypti* population dynamics. The temporal variation of the stock of individuals in each life stage (eggs, larvae, pupae and adults) is modeled as a function of the environmental carrying capacity and the climate [56]. Adaptations and simplifications are possible, such as reducing the number of vector life stages, for example considering only aquatic and winged phases. The DengueME framework allows building metapopulation models from these basic components, with easy parameterization from a geographic database. The vector model can be used alone or integrated with other models. Section 4.2 describes an example of a vector ecology model. Using a different modeling approach, vectors can be modeled as agents (for instance, considering only female adults), with individual attributes and behaviors. This strategy is used in an example presented in Section 4.3.

#### 3.2.2. Host Models

The host (human) model is another basic component that can be used by itself or as part of an integrated model of dengue. It describes the demographic dynamics of the human population in the study area. Census data can be used for the spatial allocation of individuals by age, sex or other attributes into a regular grid, defined by the user. This allocation is done by distributing the population per unit area (neighborhoods or census sectors). Using an explicit representation of space, host models can be implemented as differential equations describing the stock of individuals in each cell of the grid. An alternative is to create an individual-based model, in which each person is an agent with attributes (e.g., age, sex) and behaviors or rules (e.g., movement, susceptibility to infection). The latter method was used in the epidemic model presented in Section 4.3. Differential equations can be used to represent the human population even when an explicit representation of space is not possible (due to the lack of data) or desirable (due to the study’s goals). The transmission model presented in Section 4.1 uses this approach.

#### 3.2.3. Transmission Models

The process of virus transmission between humans and mosquitoes depends on the local amount of susceptible people (or mosquitoes) and infected people (or mosquitoes). Immune people act as barriers to transmission, since they absorb some of the bites from infected mosquitoes, without subsequently spreading the virus. Dengue models describe this dynamic process by classifying the stock of people in four states: susceptible, exposed, infected and recovered. The mosquitoes (adult females) are classified into susceptible, exposed and infected (they do not recover). There are four distinct dengue virus serotypes. If multiple viral types are being modeled, these compartments are multiplied. Some transmission models considering only one serotype were developed and are available in the framework’s library (see examples in Section 4). However, the framework supports the development of models with multiple serotypes and aspects, such as cross-immunity and variable pathogenicity [96,97,98,99,100,101,102]. Future works will include implementing multiple serotype models from the literature.

#### 3.2.4. Mobility Models

DengueME includes a mobility model, which describes the mobility and geographic commuting of humans and vectors. The spread of viruses and vectors is facilitated by the flow of individuals through air and land transportation. The human mobility component can be implemented as an unstructured model (no mobility or random mobility at a predefined intensity) or as a structured model, based on more complex structures, inspired by actual data [49]. Medeiros et al. (2011) [51] introduce distinct commuting of individuals between public and private areas for humans and smaller scale movements for mosquitoes. Mobility models for hosts and vectors, based on those described by Medeiros et al. (2011) [51], were used in the epidemic model presented in Section 4.3. Further discussions about the impact of human and vector mobility on dengue transmission can be found in [103,104].

#### 3.2.5. Landscape Class Models

Urban landscape introduces heterogeneity in *A. aegypti* breeding sites, influencing the dynamics of dengue transmission. The landscape component provides a landscape classification model to describe the space and to obtain input parameters for the models. Landscape classification can be implemented in a sophisticated way, involving segmentation and classification of high-resolution satellite images and data mining. DengueME implements a landscape classification model suited to dengue, developed by Reis (2010) [105].

#### 3.2.6. DengueME Visual Development Environment

**Figure 3:**
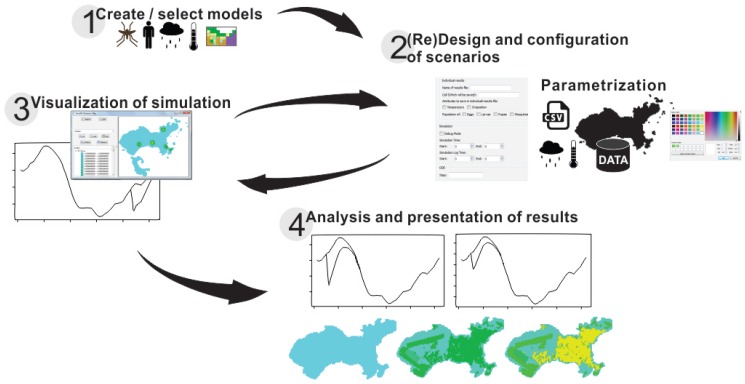
Diagram showing the modeling process using the DengueME Visual Development Environment [63].

DengueME was designed for users with no programming knowledge. The DengueME Visual Development Environment (VDE) helps users to perform the steps for developing models and defining scenarios. Its GUI provides wizards that guide users through the process, as shown in Figure 3. Users select a set of models and indicate the way they will be integrated. Then, they provide the parameters for each model and set options for output visualization and storage. User-defined settings and parameters (designed scenarios) can be stored for later use. After finishing all customization and parameterization, the TerraML source is automatically generated by the framework.

**Figure 4:**
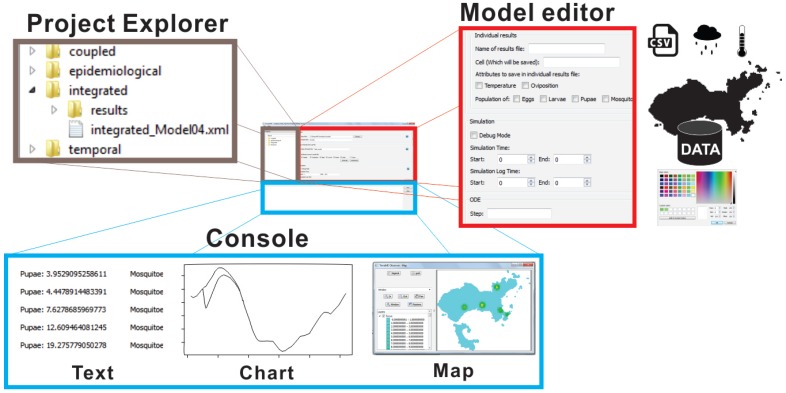
Graphical interface of the DengueME Visual Development Environment (VDE) [63].

The GUI components of DengueME VDE are presented in Figure 4. The goal is to eliminate barriers related to using programming languages and to make it easier to use the framework. Its design works with the concepts of Workspace (working directory that contains the user’s projects), View (interface components for browsing and/or presenting information on projects and models), and Editor (interface components for editing models). Figure 4 illustrates some of these features: (a) the Project Explorer displays and allows navigation through projects and existing models within the workspace; (b) the Model Editor allows setting model parameters and scenarios (e.g., using the GUI to import meteorological and other data from text files or databases) and output options; (c) the Console view presents information and views derived from the simulations.

## 4. DengueME Application

The following sections describe the pedagogical application of DengueME in three case studies. The first case study describes a simple dengue transmission model based on ordinary differential equations, proposed by Nishiura (2006) [106]. The second is an *Ae. aegypti* population dynamics model, also based on ordinary differential equations, proposed by Lana et al. (2014) [56]. The second example includes simulating the application of insecticide in some areas of a real urban space (geographic database). The third is an agent-based transmission model based on Medeiros et al. (2011) [51] and simulated in the same real urban space.

### 4.1. A Basic SIR-SI Transmission Model

**Figure 5:**
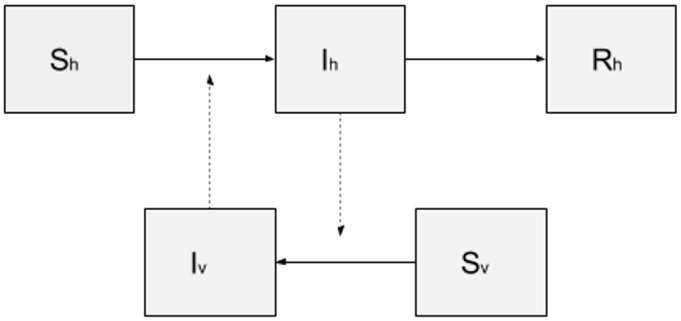
Compartmental model SIR-SI, where the human population is represented by three compartments: susceptible (Sh), infectious (Ih) and removed (Rh); and the mosquito population by two compartments: susceptible (Sv) and infectious (Iv).

The model library of DengueME offers a set of models that can be used for pedagogical purposes, to study the spatiotemporal dynamics of dengue transmission and vector ecology. As an example, a SIR-SI (Susceptible-Infectious-Removed – Susceptible-Infectious) model based on the one proposed by Nishiura (2006) [106] was developed. The conceptual model is represented in Figure 5. Human and vector populations are described using a compartmental model, where each compartment represents the amount of population in a given state (susceptible, infectious and removed). One assumption is that once a vector is infected, it remains infectious until the end of the simulation. As a simplification, the populations of humans and mosquitoes were kept constant throughout the simulation, i.e., the population dynamics of humans and mosquitoes were disregarded. The transition between states is determined by the following rates and parameters: (i) probability of transmission (vector-to-human and human-to-vector); (ii) average number of bites per mosquito per day; (iii) human recovery rate (for details about values of the parameters and equations of human and vector populations, see Nishiura (2006) [106]).

**Figure 6:**
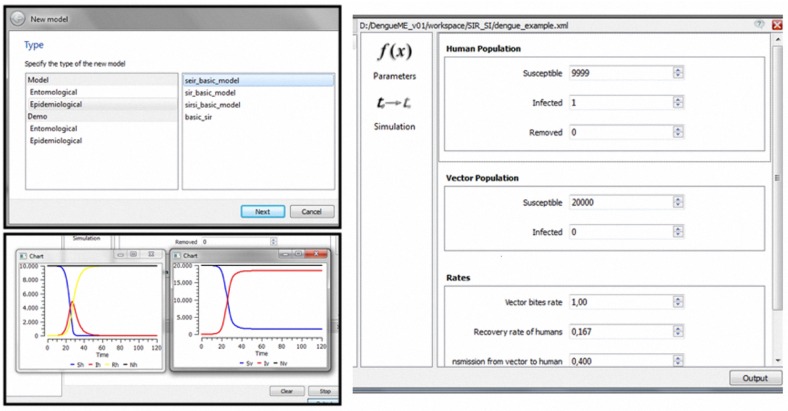
Modeling and simulation using the DengueME VDE: (**top left panel**)—Wizard to support the creation/selection of models; (**right panel**)—Graphical interface for parameterization; (**bottom left panel**)— Execution of the model and visualization of the simulation results.

Starting with this model, implemented and accessible through the DengueME VDE, it is possible to create and analyze different scenarios. A possible scenario involves seeking to understand how the parameters affect transmission dynamics. As illustrated in Figure 3, the user can first create a project and use one of the models available in the library, by interacting with “wizards” (Figure 6, top left panel). After that, the second step (Figure 3, (re)design and configuration of scenarios) involves the parameterization of the model, using a user-friendly interface (Figure 6, right panel). Some default values are provided for the parameters. Thereafter, users can run the simulation, visualize results (Figure 6, bottom left panel) and save them for further analysis.

**Figure 7:**
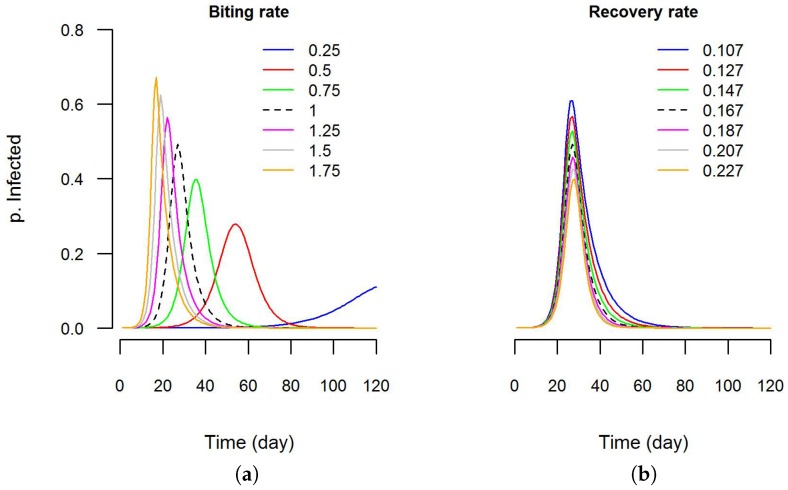
Sensitivity analysis using DengueME: (**a**) sensitivity of the predicted epidemic curve to variations in the biting rate parameter and; (**b**) recovery rate parameter. The Y-axis is the proportion infected in the human population. The black dashed line is the model output with default values (as used by Nishiura (2006) [106]).

For instance, the model was executed several times changing (one at time) the parameters “biting rate” and “recovery rate”. One of the outputs generated by the model is a CSV

file containing the values of parameters for each simulation step. This was done using the interface of Dengue VDE. Then, using the software R (Version 3.3.0) [107], graphics were created to visualize the sensitivity of the epidemic curve to these parameters (Figure 7). It is clear that the epidemic curve is more sensitive to variations in biting rate than in recovery rate. In the future, additional features (e.g., automated execution of batch simulations using value ranges for the parameters) will be developed to help users with sensitivity analyses.

The model library and user-friendly interface of DengueME support teaching-learning activities based on the creation and analysis of what-if scenarios, such as: (i) whether vector population dynamics is influenced by seasonal factors (e.g., temperature) (what happens to the transmission dynamics of dengue if the vector population increases (or decreases)?); (ii) several factors (e.g., immunity, contacts) that affect virus transmission between vectors and humans are combined into a single parameter in the model (the transmission probability). What is the sensitivity of the expected dengue dynamics to values chosen for this parameter? The parameters can be tentatively changed, and the corresponding source code of the model is automatically generated and updated, so it can be executed directly from the graphic interface. Users who have programming skills can inspect the model’s source code and adapt it manually to their own needs.

Several adaptations can be done to extend this model. For instance, another compartment could be included to represent the population (of humans, vectors or both) that is exposed to the disease (e.g., see [108,109]). Other features, like extrinsic and intrinsic incubation periods, vector ecology and explicit representation of vector life cycle phases and multiple virus serotypes, can also be included. A review of structural approaches applied to epidemiological models for dengue transmission can be found in [110].

### 4.2. Simulating the Impact of Local Interventions on Vector Density

**Figure 8:**
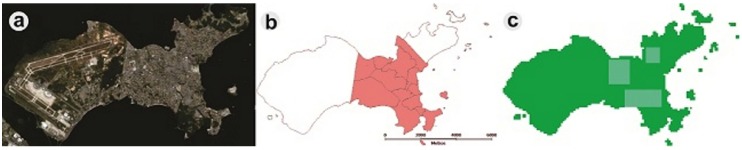
Study area, Ilha do Governador. (**a**) Satellite image (Google Earth); (**b**) map of census tracts; (**c**) simulated map generated by DengueME. Highlighted blue areas are the sites of application of adulticide [63].

An important issue is the development of standardized metrics for monitoring and evaluating the performance of control programs [48]. Alternative strategies, such as spatially-localized chemical treatments to reduce mosquito density, could be better targeted, and the resource allocation could be optimized if computer-simulated scenarios were applied. Using DengueME, we created different intervention scenarios to explore the impact of locally-applied intervention strategies on the local and global density of *Ae. aegypti*. Ilha do Governador, an island in the city of Rio de Janeiro, Brazil, was used as the study area (Figure 8). All of the stages of the vector life cycle are explicitly represented through a compartmental model and using differential equations, and the transition rates between the stages are regulated by temperature. The model and its equations are described in detail by Lana et al. (2014) [56]. A map of census tracts, obtained from IBGE (Brazilian Institute of Geography and Statistics, 2010), was used to define the regions for adulticide application, based on Luz et al. (2009) [111]. The pink area in Figure 8b was chosen to simulate the intervention, due to its denser human population. Figure 8c shows the three quadrants where the ultra-low volume adulticide, with maximum efficacy of 0.9 and an average persistence of one day, was applied.

**Figure 9:**
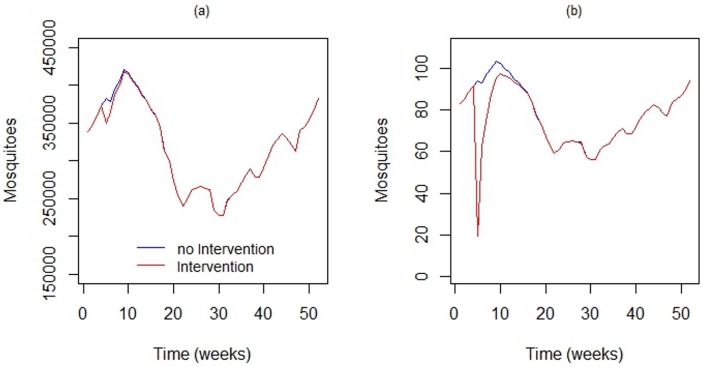
Using DengueME to simulate locally-applied chemical interventions. Comparison between the the vector population in the study area with and without the application of adulticide. (**a**) Global impact considering the entire study area; (**b**) local impact in a 100 × 100 m area [63].

Figure 9a shows the output of DengueME, showing the impact of the intervention at the scale of the whole island. Figure 9b shows the impact of this application in the local population. At the global scale, the intervention did not present a significant impact. However, at the local scale, its impact was significant for a few days. After this period, the population recovered, due to the low persistence of the simulated insecticide and the short period of application. The continuation of this work includes applying this model in different regions, using actual data, to estimate sites that require more attention from municipal epidemiological surveillance.

### 4.3. Simulating an Epidemic Scenario

**Figure 10:**
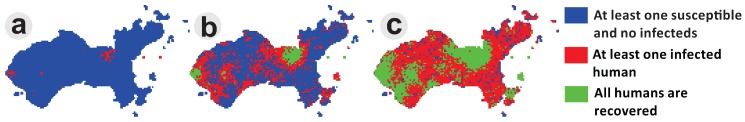
Using DengueME to simulate dengue spread from commercial to residential areas of a city. Panels show three moments of a simulated epidemic in the study area. (**a**) Beginning of the simulation with a few hotspots; (**b**) propagation waves from the hotspots; (**c**) overall dissemination and pockets of immunity [63].

The spatial spread of dengue is the result of complex dynamic interactions between humans, mosquitoes and the different virus serotypes. In this model, there are two classes of agents, humans and *Ae. aegypti* females, and only one serotype was considered. The presence of the virus in each agent is represented as human and vector attributes. Each cell corresponds to a square area unit that can represent a residential area or an area of public space. Human agents were distributed in residential areas, while vector agents were distributed across all of the cell space. Mosquitoes bite humans according to a daily biting rate, and the transmission of the virus occurs according to a given transmission probability. The mobility of mosquitoes was designed to occur locally (in the nearest neighborhood), while human mobility was designed to be concentrated in public locations, but including movements to residential areas. To illustrate this dynamic, Figure 10 shows the result of a spatial dengue spread simulation at Ilha do Governador. These simulations consider about 2500 human agents and 2000 mosquito agents. Epidemic waves travel from the commercial areas to residential areas of the island. This is an example of a dengue transmission agent-based model, adapted from Medeiros et al. (2011) [51], implemented in DengueME.

**Figure 11:**
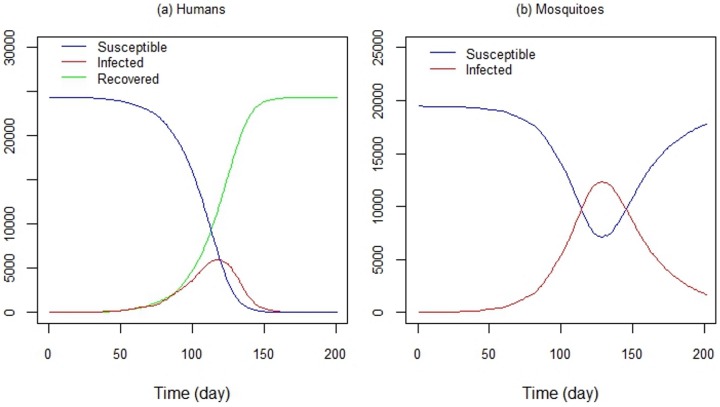
DengueME output. Time series of (**a**) susceptible, infected and recovered humans and (**b**) susceptible and infected mosquitoes, generated by DengueME simulations [63].

Figure 11a shows the time series of susceptible, infected and recovered humans, and Figure 11b shows the time series of susceptible and infected mosquitoes, respectively. Control strategies were not implemented in this experiment; only human and mosquito mobility and the virus transmission were considered. Despite this, modeling the dengue transmission dynamics is a powerful tool for evaluating control strategies, such as vaccine programs (e.g., [112,113,114,115,116,117]), as well as strategies like public education programs or the use of mosquito repellents.

## 5. DengueME Evaluation

An evaluation of the DengueME environment by potential users was carried out to identify their perception of the framework’s applicability and its usage for teaching, research and decision-making activities. The evaluation also serves as a source of information to help prioritize future development efforts. For this purpose, we conducted a group dynamic session during the III Symposium on Modeling Dengue, which took place between 8 and 10 May 2013. The group dynamic session began with a brief presentation about the framework and its objectives. Then, participants were invited to install the framework following a guide. Next, participants did an exercise parameterizing and running built-in DengueME models. At the end of the session, they were invited to fill in a questionnaire reporting their experience and providing feedback. Finally, we conducted a focus group discussion. Focus group discussions allow gathering data about participant perceptions through a moderated debate. Sixteen individuals with different backgrounds participated in the study, mostly university professors and students from several areas of expertise (entomology, medicine, epidemiology, biomedical engineering, statistics, biology, computer science, physics), with varied degrees of experience in programming and dengue modeling.

Overall, the results of this group session suggest that the DengueME VDE is friendly enough for building and running models and scenarios. All users completed the proposed tasks, most of them without any help. However, some participants reported difficulties in understanding the built-in models from the information offered by the development environment. This result indicates the need for improved documentation. Overall, we concluded that a graphical interface helps model building, and it is an important asset for fostering the use of DengueME as a tool to support the design of interventions.

**Figure 12:**
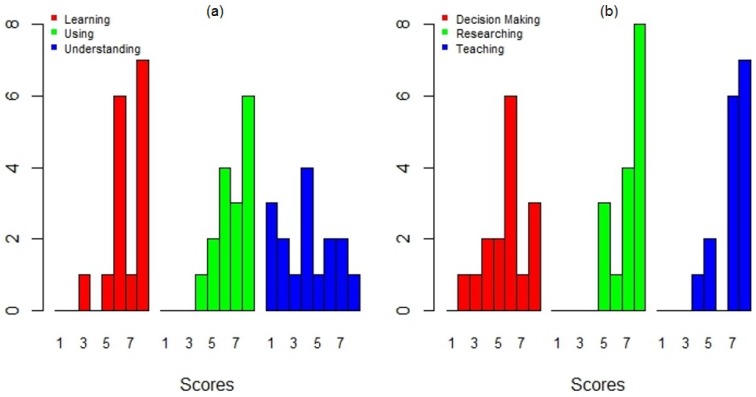
User’s evaluation of the DengueME graphical interface: (**a**) support for learning, using and understanding dengue models; (**b**) applicability potential for supporting decision making, researching and teaching [63].

An important goal of this study was to identify the applicability and potential use of DengueME in various activities. A large fraction of the participants evaluated its applicability potential for supporting research and teaching positively (Figure 12b). Some participants even demonstrated an interest in using it in class and in collaborating on the creation of new models. The participants were more divided when asked about its application as a tool to support decision making (Figure 12b). This could be explained in part by the difficulties found while using the current version, regarding the available help information and the need for users to understand the models (Figure 12a). Further effort is required to make DengueME more accessible for non-expert users (e.g., health workers).

The issue of deciding the appropriate level of detail to consider in a model depends on its purpose [62]. Some simplifications were intentionally made in the models presented in this paper to illustrate the application of the framework. There is always a compromise involving the simplicity (or complexity) level chosen during the modeling process. When deciding what level of detail is appropriate, modelers should consider factors such as intentions, understandability, availability and quality of data, explanatory and analysis capabilities with acceptable confidence degrees, levels of uncertainty and error. For instance, the introduction of a large number of parameters and behaviors, aiming to obtain a high level of fidelity to reality, may cause a computational burden. Furthermore, it can induce implementation errors and divert the attention of modelers to computational aspects, rather than letting them focus on the model itself, as a simplified representation of reality. Complexity is not an unconditionally beneficial property of a model [62]. A model should contain sufficient complexity to explain a phenomenon, but no more [88]. With models, we are trying “to gain understanding of a complex real-world system via an understanding of simpler, hypothetical system that resembles it in relevant respects” [118]. Further discussions, including philosophical ones, can be found in [62,77,118,119,120,121,122,123].

Thereby, this work described the design, development and evaluation of the tool DengueME, which aims to support the study of spatiotemporal dynamics of dengue disease. Differences in modeling paradigms, spatiotemporal scales and parameters used in the models illustrate the extensibility and flexibility of the DengueME software architecture. The visual development environment for models and scenarios was positively evaluated by a group of experts on dengue and dengue modeling. The user evaluation experience provided rich feedback to outline the next steps for DengueME. These include improvements in the mechanism for model integration through XML files and enhancements in the extensibility and usability of the software architecture.

## 6. Conclusions

Despite the success of mathematical-computational models in explaining the spatiotemporal dynamics of vector ecology and dengue transmission, it is still necessary to promote further studies and encourage their use to support planning and decision making. Models should be used in the preliminary stages of a study, during the conceptual and design phases. The proper use of these models requires understanding model assumptions. However, such use should not require extensive experience in programming techniques or in model development. Currently, fast model reuse is still a challenge.

DengueME aims to provide resources to support and encourage the study of the spatiotemporal dynamics of dengue through modeling and simulation. Its user-friendly graphical interface allows users to create and customize scenarios from built-in models, and its extensible architecture permits adding new models. By comparing the outcomes of several models, users can better understand the capabilities and limitations of each one (and of their assumptions and underlying modeling paradigm) in their attempt to reproduce the dynamics and spatial patterns observed in dengue datasets. DengueME is a tool that can reach several types of users. Teenagers in schools could learn about the epidemiology of dengue using conceptual models, varying input parameters and analyzing the outcomes. Modelers might test, adapt, extend, integrate and compare different models and modeling approaches for dengue and its vectors. Teachers of undergraduate courses could use it as a pedagogical resource in classes from different areas, such as biology, math, epidemiology, physics and computer science. Decision makers might stimulate and promote debates about dengue dynamics and public health interventions for prevention and control.

There are many initiatives to make relevant data sources public, such as temperature and maps. However, the availability and accessibility of health data is still a challenge in Brazil [124] and worldwide [27]. Efforts that aim to use data from social networks [125], news, searches [126,127,128,129,130], crowdsourcing and volunteered geographic information [131] for modeling epidemics and health surveillance are interesting and show promising results. However, such data sources should be seen as complementary or confirmatory signals to be used in models and do not replace the need for reliable public data. The lack of reliable data imposes a great difficulty for building real-time (nowcasting) epidemic surveillance systems [132] and even more for predictive and descriptive models. Efforts should be made to promote the ease of access to data, education in modeling and simulation, methods and tools with user-friendly interfaces to support (re)using, building and integrating models. Disseminating the results of analyses and predictions among the general population is potentially a tool to engage citizens in prevention and control initiatives.

This work remains under development. Partial results were obtained, analyzed and positively evaluated, motivating its continuation. Future work includes: (i) developing features for evolving and maintaining the model library (e.g., use of standardized XML schemas for model integration as provided by OpenMI, customized generation of graphical interfaces for new models, web services for software updates); (ii) performing further framework usability evaluations with potential users; (iii) developing and adding new models into the framework, such as models that represent multiple serotypes; (iv) developing new case studies; and (v) providing tutorials and demo applications to support users. Finally, we intend to extend the framework to support the modeling and simulation of other arboviruses.

## Abbreviations

The following abbreviations are used in this manuscript:

CSV: Comma-Separated Values
DengueME: Dengue Modeling Environment
DengueME VDE: DengueME Visual Development Environment
GUI: Graphical User Interface
TerraME: Terra Modeling Environment
TerraML: Terra Modeling Language
XML: eXtensible Markup Language
